# Children and young people tested for vitamin D deficiency and insufficiency at a busy children’s emergency department in Birmingham, UK: an observational study

**DOI:** 10.1136/bmjpo-2025-004311

**Published:** 2026-07-15

**Authors:** Chris Bird, Jacqueline Murphy, Qasim Malik, Philip J Turner, Gail N Hayward, Thomas R Fanshawe

**Affiliations:** 1 Emergency Department, Birmingham Women's and Children’s NHS Foundation Trust, Birmingham, UK; 2 Nuffield Department of Primary Care Health Sciences, University of Oxford, Oxford, UK; 3 Paediatrics, Birmingham Women's and Children’s NHS Foundation Trust, Birmingham, UK; 4 Department of Applied Health Sciences, University of Birmingham, Birmingham, UK

**Keywords:** Children and young people, vitamin D deficiency, diagnostics

## Abstract

**Objective:**

To evaluate the proportion of children and young people (CYP) tested in our emergency department (ED) who had either normal, insufficient or deficient serum levels of vitamin D to guide diagnostic strategies in the ED and neighbourhood outreach clinics in Birmingham.

**Design:**

Single-centre, retrospective cohort study.

**Setting:**

Children’s ED serving a superdiverse, urban population with high levels of deprivation.

**Patients and intervention:**

All CYP aged <16 years who attended the Birmingham Children’s Hospital ED and who had a serum vitamin D level sent between 1 January 2021 and 31 March 2022 were included.

**Main outcome measures:**

Primary outcome: rates of vitamin D deficiency (<25 nmol/L) and insufficiency (25–49 nmol/L) in CYP tested. Secondary outcomes: associated laboratory biomarkers; associations between deficiency/insufficiency and patient characteristics (age, gender, ethnicity, Index of Multiple Deprivation (IMD) and seasonality).

**Results:**

Of 239 CYP tested, 133 (55.6%) patients were male and mean age was 8.1 years (range 0 to 15, SD 4.8). There were 88 (36.8%) patients with vitamin D insufficiency and 62 (25.9%) who were deficient. Asian British patients had higher adjusted odds of deficiency compared with White patients (2.81 (1.11–7.85), p=0.037), but ethnicity was not associated with vitamin D insufficiency after adjustment for other patient characteristics. Increasing IMD quintile was associated with lower adjusted odds of both deficiency (OR (95% CI) per quintile: 0.59 (0.36 to 0.87), p=0.016) and insufficiency (0.58 (0.42 to 0.80), p<0.001).

**Conclusions:**

Screening is not currently advocated in the UK but we need targeted diagnostic strategies to address vitamin D deficiency in populations at greater risk.

**WHAT IS ALREADY KNOWN ON THIS TOPIC**

- National policies in both the UK and other high-income countries advise against screening children and young people (CYP) for vitamin D deficiency but an increasing number of CYP are being tested for deficiency. We have little evidence around why this is occurring but both diagnostics and supplementation policies are likely missing vitamin D deficiency in CYP from population groups at risk.

**WHAT THIS STUDY ADDS**

- We found high levels of both vitamin D deficiency and insufficiency in CYP tested in an emergency department serving a superdiverse population with high levels of deprivation. Presentations were highly heterogeneous, highlighting the challenges around diagnosis in CYP with symptoms that are often non-specific.

**HOW THIS STUDY MIGHT AFFECT RESEARCH, PRACTICE OR POLICY**

- In the absence of screening for vitamin D deficiency, this study highlights a diagnostic gap for populations at risk for vitamin D deficiency. Current approaches also likely fail to address health inequalities.

## Introduction

Vitamin D is essential to calcium and phosphorus metabolism and mineralisation of bone and is sourced mainly (80%) through ultraviolet B (UVB) light absorbed on unprotected skin which converts cholesterol to vitamin D, via hydroxylation in the liver and kidneys.^1 2^ Dietary sources of vitamin D include oily fish, eggs and liver but contribute a small proportion of required vitamin D. Vitamin D from UVB is influenced by season, latitude and skin pigmentation. Increasingly sedentary lifestyles, increased use of sun block for fear of skin cancer and clothing covering most of the body due to cultural reasons are risk factors for vitamin D deficiency.^2^ Vitamin D deficiency has gained interest not only from the re-emergence of diseases like rickets but as a prohormone for its roles in immunity, preventing inflammation, cardiovascular risk, metabolic syndrome and cancer.^1^

Reported vitamin D deficiency is increasing, from 3.14 per 100 000 person-years in 2000 (95% CI 1.31 to 7.54) to 261 per 100 000 person-years in 2014 (95% CI 241 to 281) found in a UK cohort of over 700 000 children and young people (CYP), a 15-fold increase (95% CI 10 to 21), which the study authors concluded was likely driven by increased testing by clinicians.^3^ A study in Oxfordshire evaluating trends in diagnostic tests in over 100 000 children found the largest increase in testing was for vitamin D levels (26.5% between 2005 and 2019).^4^

Vitamin D deficiency and its associated morbidity is potentially preventable and understanding and targeting testing in at-risk population groups is essential to public health and service delivery. A UK government review on intake of vitamin D is awaited^5^ and work is underway to develop strategies through fortification of food.^6^ Around 20% of UK children are thought to be vitamin D deficient, with higher rates among the South Asian and Black African Caribbean population as well as children experiencing deprivation.^7^ Appropriate targeting of vitamin supplements in children and pregnant women is a particular challenge.^8^ While most governments in well-resourced settings do not currently advocate screening for deficiency, the UK’s Scientific Advisory Committee on Nutrition recommended that ‘more sensitive biomarkers of vitamin D status and function suitable for use in population health studies and trials should be developed’.^9^ Point of care testing (POCT) would aid opportunistic and immediate counselling in at risk populations, especially in community settings and could cut healthcare costs.^10^

We aimed to evaluate the proportion of CYP tested who had either normal, insufficient or deficient serum levels of vitamin D; evaluate clinical presentations of CYP associated with vitamin D testing in our emergency department (ED) and identify risk factors for deficiency to aid improved targeting of vitamin D testing and supplementation.

## Methods

This was a single-centre, retrospective cohort study. Primary outcomes were rates of vitamin D deficiency and insufficiency in CYP tested. Secondary objectives were to: evaluate associated laboratory biomarker measurements (including serum calcium, phosphate and alkaline phosphatase levels), where available, to guide future testing strategies; evaluate associations between vitamin D deficiency/insufficiency and patient characteristics (age, gender, ethnicity and deprivation) and seasonality and summarise the presenting complaints and resulting diagnoses observed in this cohort. The study team adhered to the REporting of studies Conducted using Observational Routinely-collected health Data statement.^11^

### Population

Birmingham Women’s and Children’s NHS Foundation Trust’s busy children’s ED sees around 65 000 attendances annually and serves a superdiverse, urban population with high levels of deprivation compared with the rest of the UK.^12 13^

#### Data collection

All CYP aged <16 years who attended the Birmingham Women’s and Children’s NHS Foundation Trust’s ED and who had a serum vitamin D level sent between 1 January 2021 and 31 March 2022 were included (one patient was tested twice—we included the results for this patient as we hold they would make little appreciable difference to the overall outcomes). Testing was at clinician discretion. Samples were processed by the hospital’s biochemistry laboratory using the Elecsys vitamin D total III assay (uses a vitamin D binding protein to bind 25-hydroxyvitamin D3 and 25-hydroxyvitamin D2) on a Cobas-e analyser (Roche Diagnostics Ltd, Burgess Hill, UK).^14^

Data collection included quantitative serum 25-hydroxy vitamin D (25-OH vitamin D) levels, which were subsequently categorised in line with clinical guidelines^15^ as:

- normal: ≥50 nmol/L.
- insufficient: 25–49 nmol/L, when vitamin D supplementation is recommended.
- deficient: <25 nmol/L, when treatment is required.

We also collected quantitative, age-specific calcium, phosphate and alkaline phosphatase (ALP) serum levels when available, which were categorised (low/normal/high) from North Bristol NHS Trust Clinical Biochemistry Department).^16^ Parathyroid hormone (PTH) levels were also collected but were only available for 5% of CYP in the cohort and so were excluded from analysis (see Discussion). Patient demographic data comprised age, sex, ethnicity (defined as summarised in the online supplemental information), season, and Lower Layer Super Output Area^17^ Index of Multiple Deprivation (IMD) score (based on recorded home postcode). Presenting complaint and final diagnosis were also recorded.

Data were sourced from the hospital’s electronic pathology reporting system and the electronic patient record by CB and QM (clinicians at the hospital) who entered the data on an Excel spreadsheet on the hospital IT system, data cleaned by CB. The data were then anonymised, encrypted and sent to the University of Oxford for statistical analysis by JM and TRF.

#### Statistical analysis

##### Cohort characteristics

Characteristics of the cohort were summarised descriptively, overall and grouped by gender.

### Primary outcome: prevalence of vitamin D deficiency/insufficiency and levels of associated laboratory biomarkers

Laboratory biomarker test results were summarised descriptively using mean (SD) for quantitative results, and number and proportion of levels of categorised biomarkers (vitamin D deficiency/insufficiency and low/normal/high adjusted calcium, phosphate and ALP).

### Secondary outcomes: associations between patient characteristics and vitamin D status

Covariate-adjusted associations between patient characteristics and vitamin D status (deficient/insufficient/sufficient) were evaluated as ORs from separate multivariable logistic regression models for dichotomised levels of vitamin D status: (1) deficient versus insufficient or sufficient and (2) deficient or insufficient versus sufficient. Models were adjusted for age, gender, ethnicity and deprivation. Deprivation was summarised in descriptive statistics by quintile of IMD.

### Seasonality

The crude (unadjusted) prevalence of vitamin D deficiency/insufficiency/sufficiency was summarised by month of attendance. Subsequently, covariate-adjusted associations between season and vitamin D status were evaluated using ORs from multivariable logistic regression models, with adjustment for patient characteristics as potential confounders. For the adjusted analyses month of attendance was recoded into four seasons: Winter (December, January, February), Spring (March, April, May), Summer (June, July, August), Autumn (September, October, November).

### Presenting complaints and diagnoses

Due to the large number of reporting categories, presenting complaints and diagnoses for the cohort were summarised descriptively, both overall for the cohort and by vitamin D status.

### Treatment and supplementation

A result letter to families is standard practice at this hospital’s ED for patients with either vitamin D deficiency or insufficiency, and therefore treatment and supplementation recommendations resulting from the ED attendances were summarised descriptively for these patients.

### Missing data

There were missing data for gender/sex (6 (2.5%)), ethnicity (28 (11.7%)), and deprivation (7 (2.9%)). For patients with missing ethnicity, a separate ethnicity category (‘not reported’) was created and these patients were included in all analyses. Due to low levels of missing data, patients with missing gender/sex and deprivation were excluded from analyses requiring these data (ie, descriptive statistics stratified by age and multivariable regression analyses), resulting in complete case analyses.

### Statistical software

The analysis was conducted using R V.4.3.1^18^ and results from regression models were presented as ORs with 95% CIs.

### Patient and public involvement

The study aim and design were presented and discussed at the National Institute for Health and Care Research Community Healthcare MedTech and In Vitro Diagnostic Co-operative’s paediatric patient and public involvement group in July 2023.

## Results

The cohort comprised 239 CYP (aged between 0 and 15 years) who had attended the ED between January 2021 and March 2022 (0.3% of the total 75 862 ED attendances during the same period). One of the patients underwent testing twice.

### Cohort characteristics

133 (55.6%) included patients were male. Mean age was 8.1 (range 0 to 15, SD 4.8) years. The largest ethnicity category was Asian British, with 95 (39.7%) participants. 154 patients (64.4%) were from lowest IMD quintile (table 1).

**Table 1:** Baseline characteristics of cohort tested*

	Vitamin D status		
	Deficient	Insufficient	Sufficient
n (%)	n=62	n=88	n=89
Gender			
Female	32 (51.6)	41 (46.6)	27 (30.3)
Male	28 (45.2)	45 (51.1)	60 (67.4)
Not reported	2 (3.2)	2 (2.3)	2 (2.2)
Age			
Age <1 years	3 (4.8)	2 (2.3)	24 (27)
Age 1–5 years	6 (9.7)	20 (22.7)	27 (30.3)
Age 5+ years	53 (85.5)	66 (75)	38 (42.7)
Ethnicity			
Asian British	30 (48.4)	36 (40.9)	29 (32.6)
Black British	9 (14.5)	11 (12.5)	9 (10.1)
White	7 (11.3)	21 (23.9)	34 (38.2)
Mixed White	1 (1.6)	5 (5.7)	1 (1.1)
Any other ethnic group	8 (12.9)	4 (4.5)	4 (4.5)
Not reported	7 (11.3)	11 (12.5)	12 (13.5)
IMD quintile			
1 (most deprived)	47 (75.8)	62 (70.5)	45 (50.6)
2	9 (14.5)	13 (14.8)	21 (23.6)
3	1 (1.6)	5 (5.7)	8 (9)
4	2 (3.2)	4 (4.5)	7 (7.9)
5 (least deprived)	0 (0)	3 (3.4)	5 (5.6)
Not reported	3 (4.8)	1 (1.1)	3 (3.4)

* Gender was missing for n=6 patients.

IMD, Index of Multiple Deprivation.

#### Primary outcome: prevalence of vitamin D deficiency/insufficiency and levels of associated laboratory biomarkers

There were 150 (62.8%) patients tested with insufficient or deficient vitamin D levels: 88 (36.8%) insufficient; 62 (25.9%) deficient. Results were also recorded for a subset of patients who had additional blood tests for serum calcium, phosphate and ALP, where abnormal levels can indicate vitamin D deficiency. Among the patients with recorded test results, the majority had normal levels for each test (table 2). Of the 62 patients with vitamin D deficiency, 8 (12.9%) had hypocalcaemia; 2 (3.2%) had low phosphate levels; and 22 (35.5%) had a high ALP. Of the 88 patients with insufficient vitamin D levels, none had hypocalcaemia or low phosphate while 18 (20.5%) had high ALP.

**Table 2:** Laboratory biomarker test results by vitamin D status*

	Vitamin D status		
	Deficient	Insufficient	Sufficient
n (%)	n=62	n=88	n=89
Calcium level (nmol/L)			
Normal	32 (51.6)	61 (69.3)	64 (71.9)
Low	8 (12.9)	0 (0)	2 (2.2)
Not reported/NA	22 (35.5)	27 (30.7)	23 (25.8)
Phosphate level (nmol/L)			
High	3 (4.8)	4 (4.5)	6 (6.7)
Normal	35 (56.5)	56 (63.6)	56 (62.9)
Low	2 (3.2)	0 (0)	2 (2.2)
Not reported/NA	22 (35.5)	28 (31.8)	25 (28.1)
Alkaline phosphatase level (IU/L)			
High	22 (35.5)	18 (20.5)	23 (25.8)
Normal	19 (30.6)	41 (46.6)	47 (52.8)
Low	12 (19.4)	10 (11.4)	9 (10.1)
Not reported/NA	9 (14.5)	19 (21.6)	10 (11.2)

* Gender was missing for n=6 patients.

NA, not applicable.

#### Secondary outcomes: covariate-adjusted associations between patient characteristics and vitamin D status

In this cohort, increasing age was associated with increased odds of vitamin D deficiency (OR (95% CI) per year: 1.17 (1.08 to 1.27), p<0.001) and insufficiency (1.22 (1.14 to 1.31), p<0.001), after adjustment for other patient characteristics (table 3). Male gender was associated with reduced adjusted odds of insufficiency compared with female gender (0.43 (0.22 to 0.82), p=0.011) but was not associated with deficiency after adjustment for other patient characteristics. Ethnicity was also associated with vitamin D status; Asian British patients had higher adjusted odds of deficiency compared with White patients (2.81 (1.11 to 7.85), p=0.037), but ethnicity was not associated with vitamin D insufficiency after adjustment for other patient characteristics. Increasing IMD quintile was associated with lower adjusted odds of both deficiency (OR (95% CI) per quintile: 0.59 (0.36 to 0.87), p=0.016) and insufficiency (0.58 (0.42 to 0.80), p<0.001).

**Table 3:** Covariate adjusted logistic regression analyses for vitamin D status from complete case analyses (n=226)

	Analysis of vitamin D status adjusted for age, gender, ethnicity and IMD	Analysis of vitamin D status adjusted for age, gender, ethnicity, IMD and seasonality
	OR (95% CI; p value)	OR (95% CI; p value)
**Deficient vs insufficient or sufficient**		
Age (per year)	1.17 (1.08 to 1.27; <0.001)	1.16 (1.07 to 1.26; <0.001)
Gender (male vs female)	0.61 (0.31 to 1.19; 0.149)	0.60 (0.30 to 1.19; 0.144)
Ethnicity (Asian British vs White)	2.81 (1.11 to 7.85; 0.037)	2.81 (1.09 to 8.02; 0.040)
Ethnicity (Black British vs White)	1.83 (0.49 to 6.74; 0.360)	2.14 (0.56 to 8.12; 0.261)
Ethnicity (mixed White vs White)	1.12 (0.05 to 9.19; 0.923)	1.13 (0.05 to 9.27; 0.920)
Ethnicity (any other vs White)	6.91 (1.85 to 27.53; <0.01)	7.92 (1.98 to 34.14; <0.01)
Ethnicity (not reported vs White)	2.42 (0.70 to 8.38; 0.157)	2.77 (0.77 to 10.13; 0.117)
IMD (per quintile)	0.59 (0.36 to 0.87; 0.016)	0.59 (0.36 to 0.88; 0.017)
Season (spring vs winter)	NA	1.30 (0.54 to 3.25; 0.561)
Season (summer vs winter)	NA	0.26 (0.07 to 0.82; 0.027)
Season (autumn vs winter)	NA	0.82 (0.30 to 2.24; 0.694)
**Deficient or insufficient vs sufficient**		
Age (per year)	1.22 (1.14 to 1.31; <0.001)	1.22 (1.13 to 1.31; <0.001)
Gender (male vs female)	0.43 (0.22 to 0.82; 0.011)	0.42 (0.21 to 0.81; 0.011)
Ethnicity (Asian British vs White)	2.01 (0.91 to 4.49; 0.083)	1.92 (0.85 to 4.36; 0.115)
Ethnicity (Black British vs White)	1.97 (0.64 to 6.33; 0.243)	2.06 (0.66 to 6.77; 0.222)
Ethnicity (mixed White vs White)	7.92 (1.06 to 166.67; 0.079)	6.01 (0.81 to 126.65; 0.128)
Ethnicity (any other vs White)	2.63 (0.72 to 11.26; 0.159)	2.72 (0.71 to 12.09; 0.159)
Ethnicity (not reported vs White)	1.80 (0.66 to 5.07; 0.255)	1.88 (0.67 to 5.40; 0.232)
IMD (per quintile)	0.58 (0.42 to 0.80; <0.001)	0.59 (0.42 to 0.81; <0.01)
Season (spring vs winter)	NA	0.89 (0.35 to 2.20; 0.794)
Season (summer vs winter)	NA	0.38 (0.15 to 0.96; 0.043)
Season (autumn vs winter)	NA	0.74 (0.28 to 1.92; 0.535)

IMD, Index of Multiple Deprivation; NA, not applicable.

#### Seasonality

**Figure 1:**
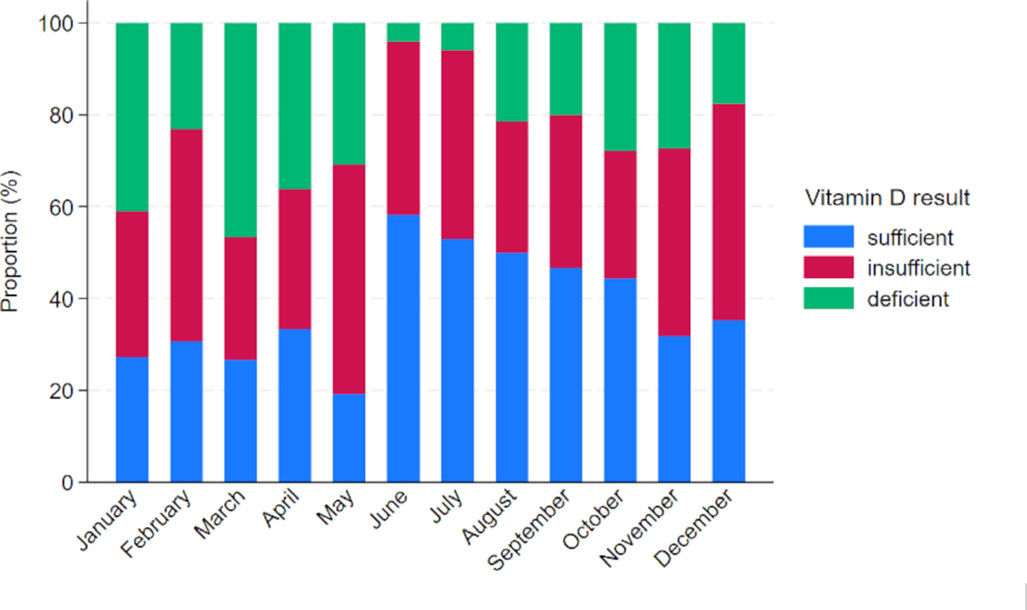
Proportion of children with vitamin D deficiency/insufficiency/sufficiency by month.

The proportion of CYP with vitamin deficiency varied by calendar month, with lower crude (unadjusted) rates of vitamin D deficiency observed in summer and autumn months, and higher prevalence in winter months (figure 1). In covariate-adjusted analyses, seasonality was associated with both vitamin D deficiency and insufficiency, after adjustment for patient characteristics (table 3). Attendances in the summer months had lower adjusted odds of both deficiency (OR (95% CI 0.26 (0.07 to 0.82), p=0.027) and insufficiency (OR (95% CI) 0.38 (0.15 to 0.96), p=0.043) compared with those in winter.

#### Presenting complaints and diagnoses

Presenting complaints for CYP that prompted testing were highly heterogeneous. The most common presenting complaints which prompted vitamin D testing (and therefore inclusion in this cohort) were limb or joint pain (54, 22.6%), abdominal pain (44, 18.4%) and fever (25, 10.5%) (see online supplemental information).

Discharge diagnoses were even more heterogeneous, with 59 different recorded diagnoses following ED consultations and biomarker testing. Vitamin D deficiency and insufficiency were the top diagnoses (30, 12.6%) followed by acute respiratory infections (26, 10.9%), limb or joint pain (18, 7.5%) and injury or fracture (17, 7.1%). Among the 150 patients with insufficient or deficient vitamin D levels, the most common diagnoses were vitamin D deficiency (30, 20%) and limb or joint pain (13, 8.7%), followed by iron deficiency anaemia (9, 6.0%) and non-specific abdominal pain (9, 6.0%) (see online supplemental information).

#### Treatment and supplementation

Following a diagnosis of either vitamin D deficiency or insufficiency (lab results at our hospital usually arrive the next day), a result letter to families with recommendations relating to further treatment for deficiency (treatment course of colecalciferol) or supplementation for insufficiency (vitamin D 400 iu daily) is standard practice at our hospital ED. Of the 62 CYP with deficient vitamin D levels, 50 (80.6%) received a letter with a recommendation for subsequent treatment but there was no letter for 12 (19.4%) patients (table 4). Of the 88 CYP with insufficient vitamin D levels, 47 (53.4%) received a recommendation for subsequent supplementation.

**Table 4:** Treatment and supplementation recommendations by vitamin D status

	Vitamin D status		
	Deficient	Insufficient	Sufficient
n (%)	n=62	n=88	n=89
Treatment			
Yes	50 (80.6)	11 (12.5)	0 (0)
No	12 (19.4)	0 (0)	89 (100)
Supplementation			
Yes	2 (3.2)	47 (53.4)	0 (0)
No	60 (96.8)	41 (46.6)	89 (100)

### Discussion

Two-thirds of CYP tested for vitamin D deficiency in our study cohort had either deficient or insufficient levels of serum vitamin D. Previous single-centre studies at UK children’s hospitals have provided data on symptomatology and demographic data^19 20^ but have not provided the proportion of children tested that were either deficient or insufficient in vitamin D. A cohort that attends hospital is not representative of the general population but the high number of children tested who had both deficiency and insufficiency is similar to data from larger studies.^321^,^23^

#### Associated blood tests

A normal POCT result for vitamin D could rationalise testing and treatment but an abnormal result would likely require further testing. UK guidance currently recommends a bone profile and PTH, along with X-rays, to check/confirm rickets and rule out secondary hyperparathyroidism (eg, in chronic kidney disease), although some hospital guidelines recommend testing PTH only if biochemistry results are atypical.^24 25^ However, given the high number of children tested who were either deficient or insufficient, the number of CYP who also had PTH tested (5%) was low for reasons that are unclear.

An observational study in Turkey found few blood test abnormalities in older children but who were symptomatic of vitamin D deficiency.^26^ Our results appear to confirm the importance of testing for vitamin D deficiency in CYP found to have a raised ALP.^27^ We were unable to evaluate additional blood test results to aid decision making around further testing as we lacked sufficient data and were unable to calculate ORs for these.

#### Presenting symptoms

Varying combinations of either vitamin D deficiency due to inadequate UVB (sunlight) exposure and/or dietary calcium intake leads to nutritional rickets, with high levels reported in the UK compared with other European countries.^28 29^ However, vitamin D deficiency is implicated in wider pathology than rickets alone.^28^

The Turkish study reported muscle weakness, low weight gain, swollen wrists and ankles in younger children while older children presented with leg and chest pain for those with deficiency.^26^ Children found to be vitamin D deficient at the Royal Hospital for Sick Children in Glasgow (n=160) had bowed legs (Ricketts, 40%), seizures (12%) and fractures (7%) at presentation.^19^ At our own institution, Kehler *et al* found in 2009–2010 the most common presentations to the ED associated with vitamin D deficiency in 89 children were abdominal pain (19%), seizures (17%) and limb pain (15%).^20^ Severe vitamin D deficiency can also cause hypocalcaemic dilated cardiomyopathy in infants.^30^

The Glasgow and Birmingham studies, both diverse cities, observed high prevalence of vitamin D deficiency in ethnic minorities with darker skin pigmentation.^19 20 30^

Symptoms of vitamin D deficiency can be non-specific and presentations in our own cohort were highly heterogeneous (see online supplemental information), with limb and abdominal pain the most common—non-specific abdominal and musculoskeletal pain are common presentations to children’s EDs.^31 32^ By combining such presentations with risk factors for vitamin D deficiency (low UVB at the UK’s latitude, darker skin tone, covered clothing), POCT could aid earlier diagnosis in at risk populations but a larger study is needed to better understand vitamin D deficiency in CYP without clinical signs of nutritional rickets.

#### Demographic and social determinants

In this study cohort, male gender was associated with decreased risk of insufficiency compared with female gender but the association was weaker for deficiency. Our findings match those of previous UK prevalence studies which found higher rates of both insufficiency and deficiency with increasing age.^3 22 23^ Absoud *et al’s* study found being overweight, increased time spent watching television and lower amounts of exercise were risk factors for vitamin D insufficiency.^22^ Our study population has high rates of obesity, while many wear clothing that covers much of the skin, both of which are associated with vitamin D deficiency and insufficiency.^33^,^35^

We also found strong associations with ethnicity, with increased risk of deficiency in Asian British and Black British CYP, matching previous prevalence studies^22 23 36^ but there was no significant difference between Asian, Black and White ethnicities for vitamin D insufficiency. Less time outdoors and more spent on screens, inadequate dietary intake of vitamin D and increased protective measures against sunlight are commonly cited reasons for vitamin D deficiency.^1^ Levels of deficiency are also affected by investment in preventive policies. A Europe-wide study found lower levels of deficiency in higher latitude Nordic countries compared with the mid-latitude UK.^37^

We also found a strong association with both season (increased risk of deficiency in the winter months) and levels of deprivation which matches previous studies^22 23^ but we need to better understand the interplay—and therefore possible confounders—between deprivation and risk factors such as ethnicity.^37^

#### Treatment

We were reliant on standard result letters as a proxy for treatment to address both deficiency and insufficiency - treatment might have been provided elsewhere. However, a fifth of CYP with deficiency and nearly a half of children with insufficiency received no letter. Evidence suggests that recommendations around supplementation are poorly adhered to^38^ and audit data from one UK children’s hospital found only two thirds of CYP in winter and a half of CYP in summer received appropriate treatment.^39^ POCT could improve communication of results and treatment.

#### Study strengths and limitations

Data were collected across all four seasons to account for seasonality alongside data on ethnicity, deprivation score, age and sex in line with previous studies in a highly diverse patient population that is at risk of vitamin D deficiency.^28^ The study also included children tested who had sufficient levels of vitamin D to better understand the proportion of children presenting to EDs who are likely to have deficient or insufficient levels which will help develop diagnostic strategies. The study also describes heterogeneous presentations which require better understanding of clinical drivers for testing.^3^

The study was a retrospective cohort presenting to hospital and so cannot represent the wider Birmingham and UK population. We did not find cases with severe sequelae of vitamin D deficiency, for example, rickets and hypovitaminosis D cardiomyopathy. We did not have enough data on associated blood tests for bone health to decide which tests might be needed in any future POCT, which should be factored into any future trial of POCT. We also had no data on lifestyle factors—diet, dress, outdoor activity—that affect vitamin D levels in children while the hospital’s paper notes did not consistently record anthropometrics. At the time of the study, we had no links to primary care data so we were also unable to more accurately determine what proportion of children received treatment/supplementation.

## Conclusions

Nearly two-thirds of CYP tested for vitamin D deficiency in our ED, which serves a superdiverse population, had either deficient or insufficient levels. Screening is not currently advocated in the UK but we need targeted diagnostic strategies, especially among CYP with non-specific presentations, to address vitamin D deficiency in populations at greater risk and underlying health inequalities.

## Supplementary material

10.1136/bmjpo-2025-004311 online supplemental file 1

## Data Availability

No data are available.
