# Supplementary material for: Children and young people tested for vitamin D deficiency and insufficiency at a busy children’s emergency department in Birmingham, UK: an observational study
Source: BMJ Paediatr Open. 2026 Jul 15;10(1):e004311. doi: 10.1136/bmjpo-2025-004311 (PMC13374397; doi:10.1136/bmjpo-2025-004311)
Supplement: online supplemental file 1 [file bmjpo-10-1-s001.docx]

**Supplementary information**

Further information on ethnicity coding

Table 1: Ethnicity grouping

| **Final ethnicity category** | **Original ethnicity text** |
| --- | --- |
| Any other ethnic group | Any other ethnic group |
|  | any other ethnic group |
|  | Mixed Any Other |
|  | Mixed- any other mixed background |
|  | Mixed any other mixed bCKGROUND |
|  | mixed other background |
|  | Other ethnic group-chinese |
| Asian British | Asian British |
|  | Asian British |
|  | Asian British Bangladeshi |
|  | Asian British Indian |
|  | Asian British- Other |
|  | Asian British- other background |
|  | Asian British Pakistani |
| Black British | Black British |
|  | Black british african |
|  | Black British African |
|  | Black British- Any other |
|  | Black British Caribbean |
|  | Black British Carribean |
| Mixed White | Mixed white and asian |
|  | Mixed White and Black African |
|  | Mixed White and Black Caribbean |
|  | Mixed white and black carribean |
|  | Mixed White Asian |
|  | Mixed White Black Caribbean |
| Not specified | n/a |
|  | not specified |
|  | [empty] |
| White | White - British |
|  | White Any Other |
|  | White any other background |
|  | White- any other background |
|  | White British |

Summary of common presenting complaints and diagnoses by vitamin D status

Table 2: Presenting complaints by vitamin D status (row percentages)

|  | **Vitamin D status** | | |
| --- | --- | --- | --- |
| **Presenting complaint** | **Deficient** | **Insufficient** | **Sufficient** |
|  | n = 62 | n = 87 | n = 89 |
| Limb, joint pain | 16 (29.6) | 24 (44.4) | 14 (25.9) |
| Abdominal pain | 15 (34.1) | 21 (47.7) | 8 (18.2) |
| Fever | 9 (36.0) | 5 (20.0) | 11 (44.0) |
| Non-specific neurological | 1 (5.6) | 6 (33.3) | 11 (61.1) |
| Injury | 2 (15.4) | 3 (23.1) | 8 (61.5) |
| Vomiting | 3 (23.1) | 6 (46.2) | 4 (30.8) |
| Weight, growth issue | 4 (33.3) | 5 (41.7) | 3 (25.0) |
| Difficulty in breathing | 1 (10.0) | 1 (10.0) | 8 (80.0) |
| Seizure | 3 (33.3) | 3 (33.3) | 3 (33.3) |
| Anaemia | 3 (42.9) | 3 (42.9) | 1 (14.3) |
| Diarrhoea | 0 (0.0) | 1 (16.7) | 5 (83.3) |
| Renal condition | 3 (60.0) | 0 (0.0) | 2 (40.0) |
| Bruising, bleeding | 0 (0.0) | 2 (50.0) | 2 (50.0) |
| Dysuria | 1 (33.3) | 0 (0.0) | 2 (66.7) |
| Limb abnormality | 1 (33.3) | 2 (66.7) | 0 (0.0) |
| Skin condition | 0 (0.0) | 0 (0.0) | 3 (100.0) |
| Lymphadenopathy | 0 (0.0) | 1 (50.0) | 1 (50.0) |
| Allergy | 0 (0.0) | 0 (0.0) | 1 (100.0) |
| Cardiac condition | 0 (0.0) | 1 (100.0) | 0 (0.0) |
| Diabetes | 0 (0.0) | 1 (100.0) | 0 (0.0) |
| Eye problem | 0 (0.0) | 0 (0.0) | 1 (100.0) |
| Lump | 0 (0.0) | 1 (100.0) | 0 (0.0) |
| Menorrhagia | 0 (0.0) | 1 (100.0) | 0 (0.0) |
| Reduced feeding | 0 (0.0) | 0 (0.0) | 1 (100.0) |

Table 3: Diagnoses by vitamin D status (row percentages)

|  | **Vitamin D status** | | |
| --- | --- | --- | --- |
| Diagnosis | **Deficient** | **Insufficient** | **Sufficient** |
|  | n = 62 | n = 87 | n = 89 |
| Vitamin D deficiency | 20 (66.7) | 10 (33.3) | 0 (0.0) |
| ARI | 4 (15.4) | 3 (11.5) | 19 (73.1) |
| Limb, joint pain | 3 (16.7) | 10 (55.6) | 5 (27.8) |
| Injury/fracture | 3 (17.6) | 5 (29.4) | 9 (52.9) |
| Non-specific abdominal pain | 1 (7.7) | 8 (61.5) | 4 (30.8) |
| Constipation | 0 (0.0) | 6 (50.0) | 6 (50.0) |
| Iron deficiency anaemia | 3 (27.3) | 6 (54.5) | 2 (18.2) |
| Renal condition | 5 (50.0) | 1 (10.0) | 4 (40.0) |
| Gastroenteritis | 1 (16.7) | 1 (16.7) | 4 (66.7) |
| PIMS TS | 3 (50.0) | 3 (50.0) | 0 (0.0) |
| Transient synovitis | 2 (33.3) | 2 (33.3) | 2 (33.3) |
| Headache | 1 (20.0) | 2 (40.0) | 2 (40.0) |
| Neurodevelopmental condition | 1 (20.0) | 1 (20.0) | 3 (60.0) |
| Appendicitis | 2 (50.0) | 1 (25.0) | 1 (25.0) |
| Asthma | 0 (0.0) | 2 (50.0) | 2 (50.0) |
| Epilepsy | 1 (25.0) | 1 (25.0) | 2 (50.0) |
| Cardiac condition | 0 (0.0) | 3 (100.0) | 0 (0.0) |
| Joint infection | 1 (33.3) | 1 (33.3) | 1 (33.3) |
| Kawasaki disease | 0 (0.0) | 0 (0.0) | 3 (100.0) |
| Crohn's disease | 0 (0.0) | 1 (50.0) | 1 (50.0) |
| Eczema | 0 (0.0) | 1 (50.0) | 1 (50.0) |
| Hypocalcaemia | 1 (50.0) | 0 (0.0) | 1 (50.0) |
| Infected eczema | 0 (0.0) | 0 (0.0) | 2 (100.0) |
| Menorrhagia | 0 (0.0) | 2 (100.0) | 0 (0.0) |
| Non-specific neurological | 0 (0.0) | 0 (0.0) | 2 (100.0) |
| Palpitations | 0 (0.0) | 0 (0.0) | 2 (100.0) |
| Perthes disease | 1 (50.0) | 0 (0.0) | 1 (50.0) |
| Rheumatology condition | 0 (0.0) | 1 (50.0) | 1 (50.0) |
| Sepsis | 1 (50.0) | 1 (50.0) | 0 (0.0) |
| SUFE | 0 (0.0) | 1 (50.0) | 1 (50.0) |
| Tumour | 0 (0.0) | 2 (100.0) | 0 (0.0) |
| Vasovagal syncope | 0 (0.0) | 0 (0.0) | 2 (100.0) |

27 diagnosis groupings with only 1 observation have been removed from the table
