# Supplementary material for: Children and young people tested for vitamin D deficiency and insufficiency at a busy children’s emergency department in Birmingham, UK: an observational study
Source: BMJ Paediatr Open. 2026 Jul 15;10(1):e004311. doi: 10.1136/bmjpo-2025-004311 (PMC13374397; doi:10.1136/bmjpo-2025-004311)
Supplement: Supplementary file 2 [file bmjpo-2025-004311.reviewer_comments.pdf]

PEER REVIEW HISTORY

BMJ Paediatrics Open publishes all reviews undertaken for accepted manuscripts. Reviewers are asked to complete a checklist review form and are provided with free text boxes to elaborate on their assessment. These free text comments are reproduced below.

ARTICLE DETAILS

**TITLE (PROVISIONAL)**	Children and young people tested for Vitamin D deficiency and insufficiency at a busy children's emergency department in Birmingham, UK: an observational study
**AUTHORS**	Bird, Christopher<br>Murphy, Jacqueline<br>Malik, Qasim<br>Turner, Philip J<br>Hayward, Gail N<br>Fanshawe, Thomas R

VERSION 1 – REVIEW

**REVIEWER NAME**	Sundaram, Premkumar
**REVIEWER AFFILIATION**	University Hospitals of Leicester NHS Trust, Paediatrics
**REVIEWER CONFLICT OF INTEREST**	No Conflict of Interest
**AI DISCLOSURE**	No, I have not used AI
**DATE REVIEW RETURNED**	16-Mar-2026

**GENERAL COMMENTS**	This is a good study evaluating vitamin D status in the heterogenous population of children and young people attending a paediatric emergency department. The paper explores relationship between vitamin D levels and important epidemiological factors like age, sex, ethnicity, socio economic deprivation and seasonality.<br>Did the authors collect additional variables like weight/height/ BMI, dietary preference (eg:vegetarian / vegan/ restrictive diet)and previous vitamin D treatment or supplementation.

VERSION 1 – AUTHOR RESPONSE

Reviewer1:
This is a good study evaluating vitamin D status in the heterogenous population of children and young people attending a paediatric emergency department. The paper explores relationship between vitamin D levels and important epidemiological factors like age, sex, ethnicity, socio economic deprivation and seasonality.

Did the authors collect additional variables like weight/height/ BMI, dietary preference (eg:vegetarian / vegan/ restrictive diet)and previous vitamin D treatment or supplementation.

These are good points but except for lab results data, which were stored in an online database, we were limited to the copied paper ED admission notes which did not record anthropometry (in a busy ED, even weight is sometimes not recorded), diet or previous Vitamin D treatment in a consistent

manner (and under Strengths and limitations, we have said that we did not address lifestyle factors). We were also limited in terms of resource as there were only two clinicians to hand search the patient records in their own time. We have added a line to the Strengths and limitations section to address this limitation.

Reviewer2:
It's a good study - single centre retrospective study, the topic is clinically relevant. Please address the following:

What were the reasons for Vitamin D testing? - was it according to clinician's discretion or did you follow any guideline for testing?

One of the principal reasons to carry out the study was to understand why CYP are tested for vitamin D deficiency at Birmingham Children's Hospital. The hospital guideline advises on the management of vitamin D deficiency but does not provide clear guidance on who should be tested. Testing is, therefore, is decided on the discretion of the clinician, with vitamin D deficiency often in the differential diagnosis for other presentations, e.g. abdominal or muscle/joint pain. I have added a short line to the Methods section (Data collection) to clarify this.

What was the proportion of children tested ? (of total ED attendances during the study period?)

The total number of ED attendances during the study period was 75,862 (1st January 2021-31st March 2022). We have added this to the results section.

There is some significant missing data - e.g. ethnicity - did you account for this in the analyses?

We have carried out extensive analysis to evaluate associations between vitamin D deficiency/insufficiency and patient characteristics (age, gender, ethnicity, and deprivation) and seasonality (see Methods, Results and Discussion). We have also summarised the presenting complaints and resulting diagnoses observed in this cohort to try and better understand what drives vitamin D testing in a children's ED.

What was the plan for those who required treatment (apart from results letter)? Do you know how many actually completed the treatment course? did you check data from primary care?

As vitamin D test results are often available only after a patient has left the ED, the only measure we have of whether or not treatment/supplementation was started is the letter to family and GP. The consultant team follow-up on all results systematically so this data is robust. However, we did not have follow-up data with either the families or primary care to know if treatment was actually started/completed. We have added a line on this in the Strengths and limitations section.

Were there any repeat attendances?

We did not look for repeat attendances as an outcome. Given the heterogenous nature of diagnoses (only a fifth of children with either deficiency or insufficiency had vitamin D deficiency as their primary diagnosis), CYP might well have re-presented for other reasons.

Was PTH not routinely tested? Is it part of your departmental Vitamin D guideline?
Please expand on other factors which may have some confounding value - sun exposure, diet etc

We are grateful for the reviewer identifying this issue as reviewing the evidence, it appears that PTH was underused (only 5% tested) given the number of CYP found to have deficient/insufficient levels of vitamin D. Other hospitals (e.g. Alder Hey Children's Hospital, Melbourne Royal Children's Hospital) advise that PTH is not routinely tested, with this test reserved for atypical results when first-line test results are atypical. We have addressed this in the Discussion (Associated blood tests) and also hypothesised that a lack of understanding around rational testing and/or assumptions that deficiency/insufficiency were nutritional/environmental.

Can you ensure the manuscript clearly distinguishes prevalence in tested patients compared to population prevalence ?

Thank you for making this important point. I have rephrased some sentences in both the Results and Discussion section, removing the word "prevalence", to clarify that the proportions relate to the CYP tested and not the total hospital population.
